# MiR-133b Regulates the Expression of the Actin Protein TAGLN2 during Oocyte Growth and Maturation: A Potential Target for Infertility Therapy

**DOI:** 10.1371/journal.pone.0100751

**Published:** 2014-06-24

**Authors:** Guohong Xiao, Chenglai Xia, Jie Yang, Jianqiao Liu, Hongzi Du, Xiangjin Kang, Yuyi Lin, Ronghua Guan, Pengke Yan, Shengsong Tang

**Affiliations:** 1 Department of Pathology, University of South China, Hengyang, China; 2 The Third Affiliated Hospital of Guangzhou Medical University, Guangzhou, China; 3 Guangdong Provincial Key Laboratory of Reproductive Medicine, Guangzhou, China; 4 Center for Life Science, Hunan University of Arts and Science, Changde, China; Shanghai Medical College, Fudan University, China

## Abstract

Infertility is an area of increasing in life science research. Although follicular maturation disorders and anovulation are the primary causations of infertility, its molecular mechanism is not well understood. Recent research has shown that microRNAs (miRNAs) might play an important role in the regulation of ovarian follicle development and maturation. In this study, the expression of miRNAs in metaphase I (MI) oocytes treated with or without insulin-like growth factor 1 (IGF-1) was observed by microRNA microarray analysis. Results show that 145 miRNAs were up-regulated and 200 miRNAs were down-regulated in MI oocytes after IGF-1 treatment. MiR-133b, which was up-regulated more than 30-fold, was chosen for further research. As a potential target of miR133b, *transgelin 2* (*TAGLN2*) gene was down-regulated, at both transcription and translation levels, in miR-133b- over-expressed 293T cells, but *TAGLN2* was up-regulated when the expression of miR-133b was inhibited. Furthermore, the expression level of *TAGLN2* in the ovaries of 8-week- old mice was higher than that observed in 4-week-old mice. Immunofluorescence experiments showed that *TAGLN2* was located in the cytoplasm. In general, our results indicate that miR-133b may play important roles in the growth and maturation of oocytes by regulating its potential target, *TAGLN2*, at both transcription and translation levels. Therefore, our research provides a potential new target for infertility therapy.

## Introduction

For sustained and successful reproduction, proper development and function of the hypothalamic-pituitary-gonadal axis and its regulation of the cervical, uterine, and oviductal tissues are imperative. Follicular development and ovulation are very complicated processes, including changes of follicle morphology, endocrine regulation, as well as local factors and genes, at different periods. Hormone secretion, growth factor release, and gene expression must be modulated accurately, both temporally and spatially, to ensure proper growth and development of oocytes and ovulation.

Gene expression can be regulated at both transcription and translation levels. MicroRNAs (miRNAs) are a class of single-stranded, endogenous, non-coding small (18–24 nucleotides) RNAs that participate in gene expression regulation at different levels. It has been reported that miRNAs participate in the regulation of cell proliferation, differentiation, apoptosis, migration, tissue inflammation, tumor formation and energy metabolism at different levels, including transcription, translation, and post-translation [1]–[4]. The roles of miRNAs in regulating ovarian functions were first expounded by Otsuka and his colleagues who used targeted deletion of the *Dicer1* gene (*Dicer1*d/d) in mouse [5]. Dicer1 functions as a ribonuclease and is required by the RNA interference and microRNA pathways to produce the active small RNA component that represses gene expression. *Dicer1* d/d ovaries transplanted into the wild-type female mouse led to infertility, but transplantation of wild-type mouse ovary into Dicer1d/d females resulted in offspring, suggesting that this fertility defect was derived from the ovary [6]. Dicer1 knockout oocytes also led to the loss of oocyte meiotic spindle structure and chromosome condensation, in turn, resulting in the loss of meiotic division follicular developmental abnormalities, ovulation disorders, apoptosis and infertility [7]–[9]. Such *dicer1* mutants indicate that miRNAs play important roles in fertility by regulating follicular and ovarian functions. The expression of miR-103, miR-16, miR-30b, miR-30c and let-7d played an important role in maintaining the stability of the spindle structure [10]. Pri-miR21 expression increased by 30 times in the theca cells of immature mice after 4 h of human chorionic gonadotropin (hCG) stimulation, while miR-21 increased by 5.8 times after a 6 h hCG treatment in mature mice. Injecting miR-21-LNA into the ovarian bursa led to obvious change in the morphology of granulosa cells, and miR-21 expression was up-regulated, while the degradation of caspase-3 was reduced under luteinizing hormone (LH) treatment. Meanwhile, an increased apoptosis of granulosa cells and decreased ovulation rate occurred in the ovary of miR-21 knockout mice [11]. Previous research reported that miR-181a expression was down-regulated in a dose- and time-dependent manner under activin A treatment in mouse granulosa cells (mGC). Additionally, over-expression of miR-181a down regulated cyclin D2 and PCNA expression by binding to the target gene *acvr2a* in the granulosa cells, resulting in the inhibition of cellar proliferation [12]. Another study demonstrated that miR-224 was a potential mediator regulating the TGF-β signaling pathway by targeting Smad4 in mouse pre-antral granulosa cells, which may promote CYP19A1 gene expression and 17β-estradiol secretion from pre-antral granulosa cells [13]. Furthermore, in the granulosa cells, the tumor suppressor gene p53 and NF-κB p65 cooperated to inhibit miR-224 expression, resulting in the proliferation of granulosa cells and estradiol release [14].

The IGF-1 signaling pathway plays an important role in the complex process of follicular development with hCG treatment [15]. Bachelot et al. reported that ovarian follicular growth stopped in IGF-1 KO mice [16]. Therefore, IGF-1 was speculated to regulate the growth of oocytes and the development of primary follicles from primordial follicles. Additionally, IGF-1 stimulates granulosa cell proliferation, increases estrogen and progesterone synthesis, and improves aromatase activity and amino acid accumulation *in vitro* [17]. IGF-1, which synthesizes in granulosa cells and exists in follicular fluid, inhibits cell apoptosis and promotes follicular maturation with the assistance of gonadotropin [18]. Finally, IGF-1 acts as a hub, connecting multiple signaling pathways into a network which regulates follicular growth, development and maturation.

Here, our results indicate that 145 miRNAs were up-regulated and 200 miRNAs were down-regulated in MI oocytes after IGF-1 treatment. MiR-133b, which was up-regulated more than 30-fold, was found to have a putative target, *transgelin 2* (*TAGLN2*), which encodes an actin protein, TAGLN2. TAGLN2 protein is widely present in mammalian smooth muscle tissues [19]. MiR-133b may therefore play important roles in oocyte growth and maturation by negatively regulating its potential target *TAGLN2* at both transcription and translation levels.

## Results

### miRNA microarray analysis

**Figure 1:**
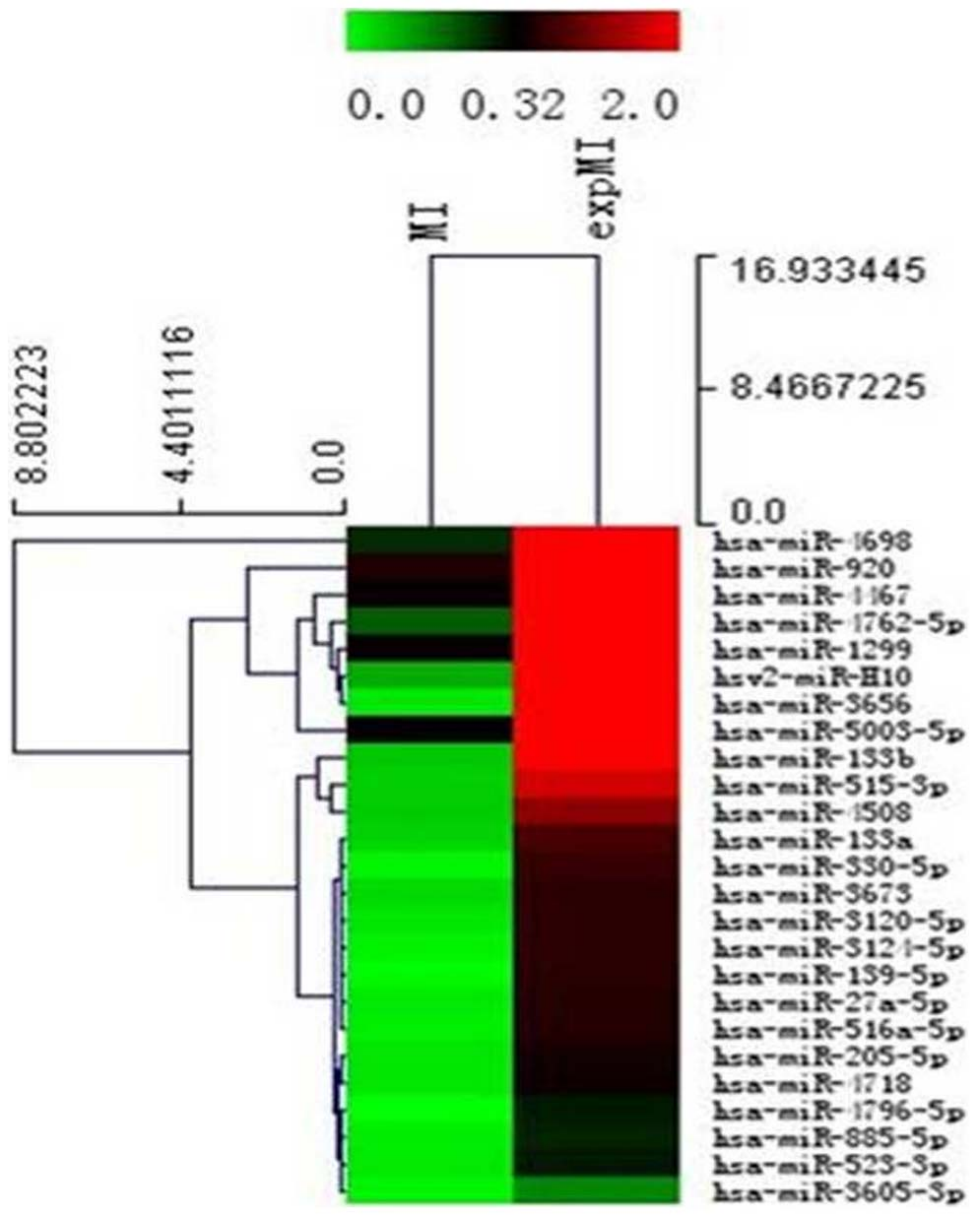
Hierarchical clustering of miRNAs deregulation. The miRNAs were degraded >2-fold in MI oocytes with and without IGF-1 treatment. MI oocytes cultured at 37°C and 5% CO_2_ were treated with or without 200 ng/ml IGF-1, after 24 h, all oocytes were stored at 80°C until RNA was extracted for microarray analysis. The color bar indicates that miRNA expression levels increased from green to red compared with controls(n = 3, mean±SD).

To ensure the high quality of our results, significant changes in miRNA levels (>2-fold) were identified in MI oocytes disposing in the IGF-1, compared with the negative control group. As shown in the heat- maps, 145 miRNAs were upregulated, and 200 miRNAs were downregulated (Figure 1). Of these miRNAs, the relatively prominent upregulated miRNAs were hsa-miR-3656, hsa-miR-139-5p, hsa-miR-4796-5p, hsa-miR-330-5p, hsa-miR-4698, hsa-miR-3124-5p, hsv2-miR-H10, hsa-miR-133b, hsa-miR-515-3p, hsa-miR-516a-5p, hsa-miR-4762-5p, hsa-miR-4508, hsa-miR-27a-5p, hsa-miR-3120-5p, hsa-miR-133a, and hsa-miR-205-5p (>15-fold), and the relatively prominent downregulated miRNAs were hsa-miR-411-3p, hsa-miR-19b-3p, hsa-miR-152, and hsa-miR-142-5p (>15-fold).

### Subcellular location of TAGLN2

**Figure 2:**
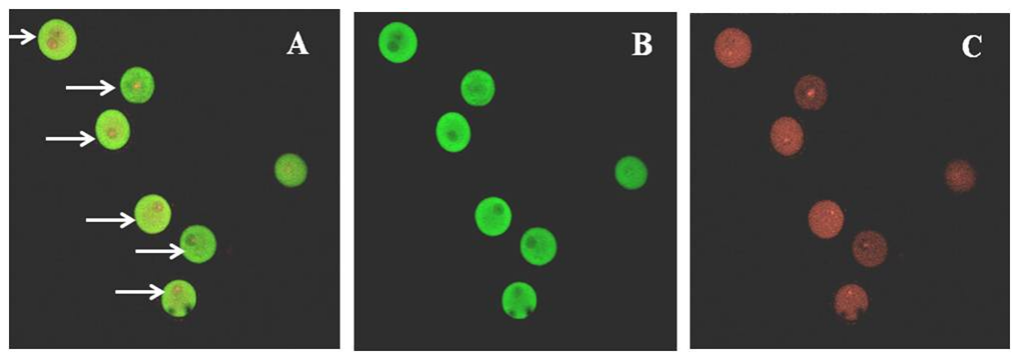
The subcellular location of TAGLN2 was measured by immunofluorescence. TAGLN2 was labeled with green fluorescence, while cell nuclei and cell membrane were shown as red fluorescence. A)merge; B)Green fluorescence; C)Red fluorescence(10×20, n = 3).

We analyzed the subcellular location of TAGLN2 by immunofluorescence. Cells were first fixed, then blocked by goat serum, and, finally, incubated with primary antibody and FITC-labeled secondary antibody in order, followed by analysis using confocal laser scanning microscopy (CLSM). As shown in Figure 2, TAGLN2 was labeled with green fluorescence, while cell nuclei and cell membrane were shown as red fluorescence. Based on this evidence, it was concluded that TAGLN2 is expressed in cytoplasm, but not cell nuclei.

### Expression of TAGLN2 in mouse ovary and human oocyte

**Figure 3:**
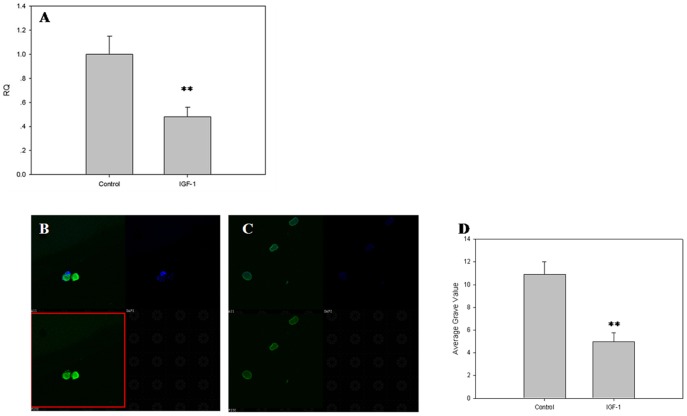
The expression of TAGLN2 at the human oocyte. After treatment with IGF-1 for 24 hours, the expression levels of *TAGLN2* in human ovary were analyzed by real-time PCR and immunofluorescence: A) The expression levels of *TAGLN2* in human ovary; B) The expression levels of TAGLN2 in human ovary; C) The expression levels of TAGLN2 in human ovary after treatment with IGF-1; D) Average gray value. Each sample was tested in triplicate and the data were presented in mean ± SD. The experiments were repeated twice and similar results were obtained (mean±SD, n = 3, **P<0.01).

After treatment with IGF-1 for 24 hours, the expression levels of *TAGLN2* in human oocyte were analyzed by real-time PCR and immunofluorescence. The *TAGLN2* mRNA and TAGLN2 protein expression level were just half that of control post-treatment. This finding suggested that IGF-1 downregulates *TAGLN2* in human ovary, in turn, suggesting that *TAGLN2* might be the downstream gene in the IGF-1 signaling pathway (Figure 3A–D).

**Figure 4:**
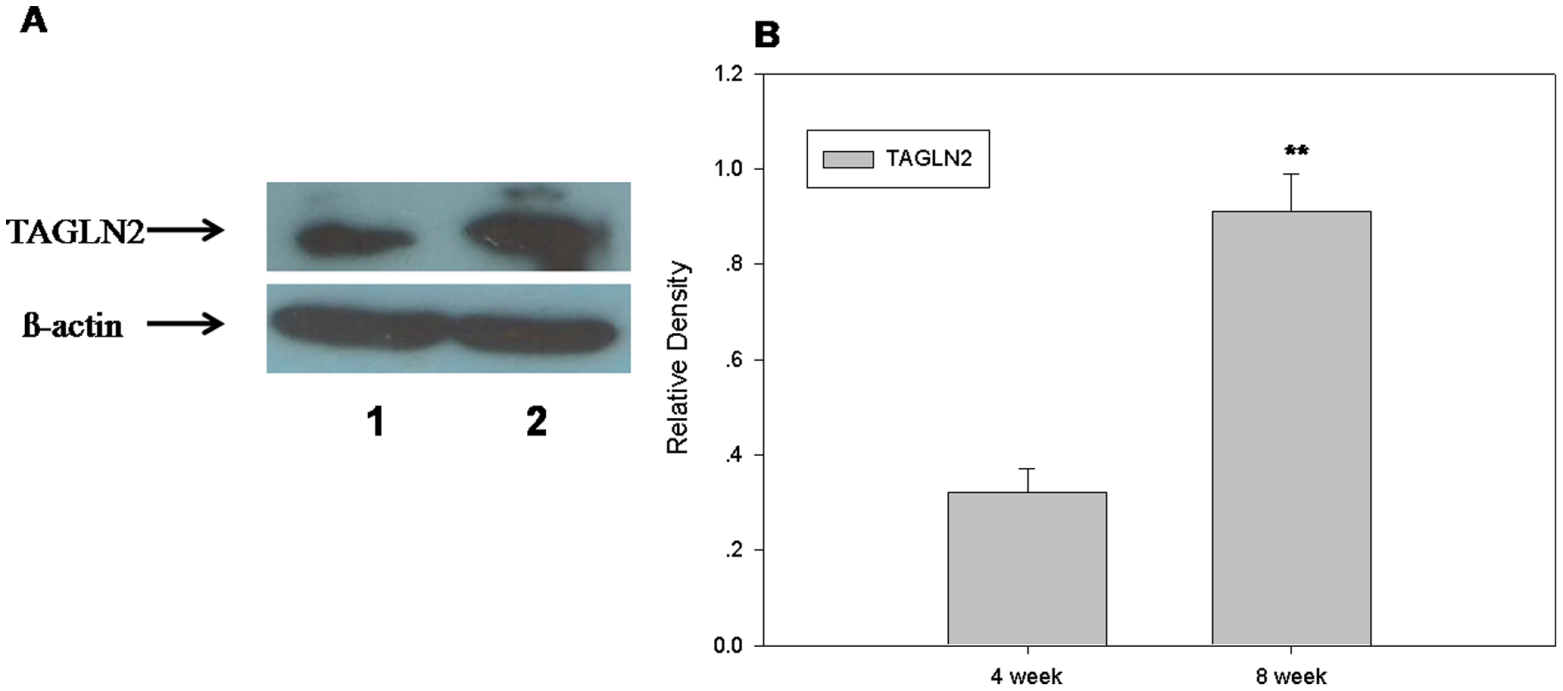
Expression of TAGLN2 in mouse ovarian. Western blotting was used to measure the expression of TAGLN2 protein in mouse ovary. TAGLN2 had a higher expression level in the 8-week-old mouse ovary than that of the 4-week-old mouse ovary.A)Westernblot:1. 4-week mouse's ovarian, 2. 8-week mouse's ovarian and B) Relative Density(n = 3, mean±SD, **P<0.01).

Western blotting was used to measure the expression of TAGLN2 protein in mouse ovary. As shown in Figure 4, TAGLN2 had a higher expression level in the 8-week-old mouse ovary than that of the 4-week-old mouse ovary. This suggests that *TAGLN2* may participate in ovarian development and maturation.

### Putative targets of miR-133b

**Figure 5:**
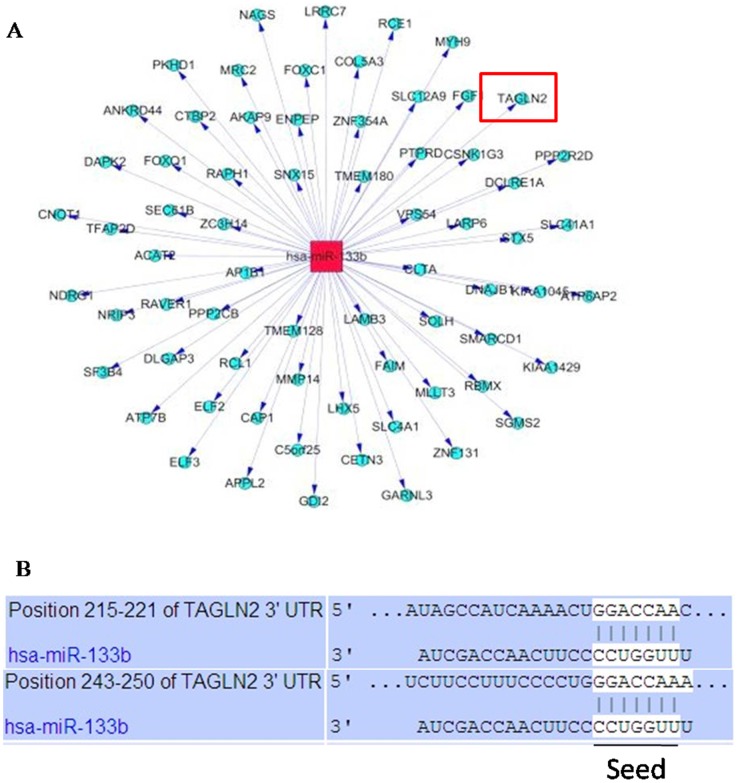
Target genes of miR-133b. Three algorithms DIANA, TargetScan 4.0 and PicTar were all used for the prediction of the targets. Only the targets identified by all of them were shown Figure 5A. 168 putative miRNA targets were identified by the Three algorithms. The detail acting site for hsa-miR-133b binding to TAGLN2 was shown Figure 5B. Red rectangle represents miR-133b, and blue circle nodes represent mRNAs.

In total, 345 differentially expressed miRNAs were identified in our miRNA microarray analysis. Hsa-miR-133b, one of the highest upregulated miRNAs (32.74-fold), has not previously been reported as playing a role in follicle growth and ovulation. Therefore, it was selected to predict potential targets and functions. Three algorithms, including DIANA, TargetScan 4.0 and PicTar, were all used for the prediction of the targets. Only the targets identified by all three algorithms were analyzed further. As shown in Figure 5, 68 putative target mRNAs of miR-133b were found.

### miR-133b modulates *TAGLN2* in 293T cells

**Figure 6:**
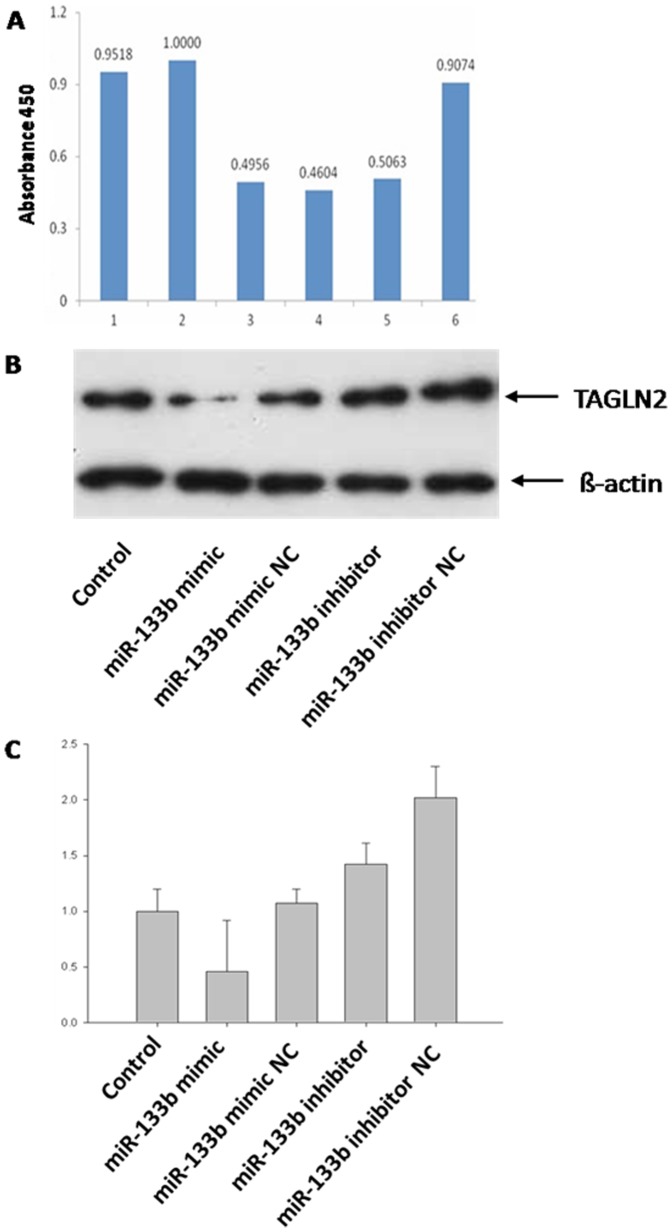
miR-133b targets TAGLN2 in 293T cells. A) Vertical axis is the ratio of Renilla to firefly luciferase and the horizontal axis is the experiment groups: 1. siCHECK-NP; 2. siCHECK-3UTR+microRNA-NC; 3. siCHECK-3UTR+25 nM microRNA-133b; 4. siCHECK-3UTR+50 nM microRNA-133b; 5. siCHECK-3UTR+100 nM microRA-133b; 6. siCHECK-3UTR-m+100 nM microRNA-133b. B) Protein expression of TAGLN2 in 293T cells after being treated with miR-133b. C) mRNA expression of TAGLN2 in 293T cells after being treated with miR-133b. Each sample was tested in triplicate and the data were presented in mean ± SD. The experiments were repeated twice and similar results were obtained. **P<0.01.

The putative target gene analysis indicated that miR-133b may regulate *TAGLN2* (Figure 6) which has been reported as having a potential oncogenic function [20]. To demonstrate whether miR-133b can modulate *TAGLN2* expression at different levels, miR-133b mimic, miR-133b inhibitor, and their negative controls were transformed into human 293T cells. The expression of *TAGLN2* at mRNA and protein levels was then detected by real-time PCR and Western blotting, respectively. *TAGLN2* was significantly down-regulated, both at mRNA and protein levels, in 293T cells transformed with miR-133b mimic compared to the control cells and miR-133b mimic NC cells (Figure 6 B, C). In contrast, both mRNA and protein level of TAGLN2 in 293T cells transformed with miR-133b inhibitor were higher than seen in control cells (Figure 6 B, C). These results suggested that TAGLN2 protein level was negatively regulated by miR-133b at both transcription and translation levels. However, mRNA and protein levels of TAGLN2 in 293T cells transformed with miR-133b inhibitor were lower than those of 293T cells transformed miR-133b inhibitor negative control (NC), but we are unable speculate about the reasons for this phenomenon.

Based on our algorithms, the 3′UTR sequence of *TAGLN2* was believed to be the target of miR-133b. To further investigate how miR-133b regulates *TAGLN2*, we constructed the 3′UTR sequence of *TAGLN2*, or the mutation sequence, into the Renilla luciferase reporter system, and the inserted sequence was contained in the reporter gene mRNA. All the Renilla luciferase reporters were injected into 293T cells with or without miR-133b at the same time. For the reporter containing *TAGLN2*-3′UTR expressed without miR-133b, reporters were expressed at the same level as control cells; the reporter containing *TAGLN2*-3′UTR mutation was expressed with 100 nM miR-133b. However, all the levels of reporters were approximately half that of control such that the reporter containing *TAGLN2*-3′UTR was expressed with 25, 50, or 100 nM miR-133b, respectively (Figure 6A). The results showed that miR-133b may inhibit the expression of *TAGLN2* by combining with its 3′ UTR directly in a non-dose-dependent manner.

## Discussion

Follicle development is a very complicated, but precise, process. Oogenesis starts from the formation of the primordial germ cell. Oocyte starts meiosis phase I at the very beginning of DNA synthesis, and oocytes stay in prophase I until the resumption of meiosis, because of the influence of multiple inhibitory factor, such as cAMP [21]–[22]. Before ovulation, oocytes become secondary oocytes after completing the first meiosis and stop at metaphase II of meiosis until fertilization. These processes are regulated by various factors, including sex hormones and intrinsic factors, such as IGF and TGF-β. Recently, miRNAs were reported to be involved in the regulation of the female reproductive process, or, more specifically, the development of reproductive organs at specific stages. As such, the specific effects of miRNAs on the development and maturation of oocytes have attracted considerable research attention. In particular, researchers have asked if miRNAs participate in the IGF-1 signaling pathway, which regulates follicle development.

Recently, many miRNAs have been predicted to regulate follicle development. Murchison et al. first investigated the expression of miRNAs in mouse oocytes, and they demonstrated that the miR-30, miR-16 and let-7 family was overexpressed in mouse germinal vesicle (GV) oocytes, speculating, as a result, that miRNAs might play important regulatory roles in the expression of mRNAs during the process of follicular maturity [23]. Furthermore, Tang et al reported that the miR-30, miR-16, let-7 and miR-17-92 family, which was detected in mature mouse oocytes, dynamically regulated oogenesis and early embryonic development. They also found that the miR-17-92 family of miRNAs inherited from fertilized egg increased in oogenesis, and increased higher after the two-cell stage of embryo [24]. Thus, it has been reported that miRNAs directly or indirectly regulate the expression of some maternal genes at the post-transcriptional level and may, furthermore, take part in the early stage of follicle development.

Cell-cell communication plays an important role in follicle growth and development, and exosomes were reported to be participants. It was reported that 509 and 356 miRNAs were found in the exosomes and non-exosomes, respectively, isolated from bovine follicular fluid. Of these miRNAs, 331 were detected in both exosomes and non-exosomes simultaneously [25]. In microvesicles and exosomes from equine follicular fluid, 79 miRNAs and 41 miRNAs were detected, respectively, and 22 and 35 miRNAs were detected in the granulosa cells and cumulus cells, respectively [26]. MiRNAs encompassing the oocytes may therefore modulate the growth and maturation of oocytes by facilitating cell-cell communication.

miRNAs modulate the growth and maturation of follicle and oocyte by various signaling pathways. A recent study on miRNA expression in the ovaries of 3- and 5-day-old mice showed that 15 miRNAs were upregulated, and 9 miRNAs were downregulated in 5-day-old mouse ovaries compared with the 3-day-old mice. Also, the miR-145 antagomir decreased the number of primordial follicles, but increased the growth of follicles *in vitro* by upregulating TGFBR2 and activating SMAD3 of the Smad signaling pathway, which induces the apoptosis of primordial follicles [27]. According to the miRNA profiles, mmu-let-7b, mmu-let-7c, mmu-miR-27a and mmu-miR-322 were significantly downregulated in the granulosa cells of mouse MII oocytes compared with those of MI oocytes. Oocytes matured more slowly by injecting mmu-miR-27a mimic into granulosa cells, but faster by injecting mmu-let-7c-, mmu-miR-27a- and mmu-miR-322-inhibitor, compared with the negative control group. These miRNAs may therefore participate in the meiosis of oocyte *in vitro* maturation [28].

The expression of miRNAs is spatial and temporal during the development and maturation of human oocytes. Among 722 miRNAs, 4 miRNAs were upregulated and 9 were downregulated in the MII oocytes compared with the GV oocytes [29]. Moreover, analysis of the miRNA expression profiling data and the list of target mRNAs showed that miR-100, miR-184 and miR-10a were especially expressed in human MII oocytes, while miR-29a, miR-30d, miR-21, miR-93, miR-320a, miR-125a and let7 were expressed in the human cumulus cells. These miRNAs have various biological functions, including regulation of gene transcription, cell cycle, oocyte reprogramming, and anti-apoptosis, among others [30]. These findings suggest that specific miRNAs may play a specific role in the regulation of human oocytes and cumulus cells by their regulation of target genes.

In order to further understand the molecular mechanism underlying the activity of miRNAs in the IGF-1 signaling pathway relative to the regulation of follicle and oocyte development, we investigated the expression of miRNAs in MI oocytes treated by IGF-1 using the microRNA microarray. A total of 345 differently expressed miRNAs were identified, including 145 upregulated miRNAs and 200 downregulated miRNAs in the MI oocytes after IGF-1 treatment compared with MI oocytes without IGF-1 treatment.

In order to demonstrate the specific regulatory functions in the oocytes, the highly (32.74-fold) induced miR-133b was chosen to predict target genes. The human miR-133b is located at chromosome 6p12.2, which was initially thought to be a muscle-specific miRNA and, hence, upregulated during muscle development [31]–[32]. Subsequent studies have found that miR-133b could be widely detected in many tissues. According to our miRNA profiling, miR-133b was upregulated in MI oocytes treated with IGF-1 compared with the negative control. Recently, miR-133b was found to inhibit the expression of Foxl2 in human and mouse granulosa cells by binding to the Foxl2-3′ UTR, but increase the expressions of StAR and CYP19A1 to promote estrogen secretion in granulosa cells simultaneously [19]. Furthermore, analysis revealed that *TAGLN2* was a target gene of miR-133b. *TAGLN2* is expressed in many tissues, including bladder, stomach, liver, lung, prostate, uterus and ovaries, and it could promote cell apoptosis. In our study, the expressions of *TAGLN2* mRNA and protein were significantly reduced after being injected with miR-133b mimic into 293T cells, but were increased with miR-133b inhibitor, compared with the control group. Moreover, miR-133b directly targeted TAGLN2-3′ UTR to reduce *TAGLN2* at both transcriptional and translational levels.

It was reported that *TAGLN2* is expressed in uterine luminal epithelium, glandular epithelium, endometrial and embryonic implantation sites and that it participates in the regulation of embryo development and the occurrence, invasion, metastasis and differentiation of endometriosis [33]–[35]. Additionally, the expression of *TAGLN2* was higher in the *in vivo* maturation of bovine cumulus cells compared with *in vitro* maturation [36]. We confirmed that the level of *TAGLN2* is higher in the 8-week-old mouse ovary compared to that of the 4-week-old mouse ovary, suggesting that TAGLN2 is, in fact, present in the ovary and that it may participate in the regulation of follicular and ovarian development and maturation.

In summary, our data indicated that miRNAs can be deregulated by IGF-1 in human MI oocytes. MiR-133b was highly up-regulated and inhibited the expression of *TAGLN2* directly by targeting *TAGLN2*-3′ UTR. This suggests that miR-133b and *TAGLN2* may be the downstream genes as a part of the IGF-1 signaling network. However, the molecular mechanisms of miR-133b and *TAGLN2* in the growth and development of human oocytes need further research. Nonetheless, the findings in the present study will provide a sound theoretical basis for explaining oocyte development and will generate new ideas for improving the possibility of successful embryo culture and embryo development *in vitro*, as well as new strategies for infertility therapies.

## Materials and Methods

### Ethical approval

After the informed consent approval was obtained, the oocytes were collected from the patients who were explicitly informed about the research aims, their rights and interests in the research. From April to July 2013, a total of 107 oocytes at MI stage were obtained from patients who received intra-cytoplasmic sperm injection (ICSI) treatments at the Reproductive Medical Center of the Third Affiliated Hospital of Guangzhou Medical University. This study and the use of patients' oocytes were approved by the Ethics Committee of the Third Affiliated Hospital of Guangzhou Medical College. All procedures complied with the Declaration of Helsinki and written consent was obtained from all patients who received ICSI treatments. The ethics committee approved this consent procedure.

ICR mice were obtained from the Animal Center of Guangdong Province. This study was approved by the Ethics Committee of the Third Affiliated Hospital of Guangzhou Medical University.

### Reagents and cell culture

The isolated MI oocytes were cultured at 37°C and 5% CO_2_ in G-2 PLUS (Vitrolife, Frölunda, Sweden) treated with (45 oocytes) or without (62 oocytes) 200 ng/ml IGF-1 (Peprotech, Rocky Hill, NJ, USA) for 24 h, then covered with ovoil (Vitrolife). After 24 h, all oocytes were stored at 80°C until RNA was extracted. The average age of patients without IGF-1 treatment was 31.66±3.90. The average age of patients with IGF-1 treatment was 31.25±3.31. No apparent bias was detected between these two groups.

The 293T cells injected with MiR-133b mimic, MiR-133b mimic negative control, MiR-133b inhibitor, or MiR-133b inhibitor negative control (NC) (Ribobio, Guangzhou, China) were cultured at 37°C and 5% CO_2_ in DMEM (Gibco, Grand Island, USA) and supplemented with 10% fetal bovine serum and 1% antibiotics (100 U/ml penicillin and 100 U/ml streptomycin.

### MicroRNA microarray analysis

Denuded oocytes were stored in Trizol at −80°C prior to RNA purification. RNA from 45 oocytes in each treatment as above was pooled. To satisfy the minimum requirement of 250 ng RNA for microarray analysis, 300–400 ng RNA were used for each analysis. In total, 6 microarray analyses, including three repeats for each treatment, were performed.

MicroRNA expression was surveyed by using the mercury LNA microarray platform (Exiqon, Denmark). All procedures were carried out according to the manufacturer's protocols. Briefly, miRNAs were enriched from extracted total RNAs using RNasey Mini Kit (Qiagen p/n 74104) and labeled with miRCURYTM Array Power Labeling kit (Exiqon, Denmark). Labeled miRNAs were used for hybridization on a miRCURY LNA microarray (v.10.0-hsa, mmu & rnoarray) containing 345 human miRNAs corresponding to all microRNAs annotated in miRBase 10.0, as well as viral microRNAs (http://microrna.sanger.ac.uk).

Following hybridization, arrays were stained, washed, and scanned using an Axon GenePix 4000B microarray scanner and Genepix pro v6.0 software. Four replicate spots of each probe on the same slide were averaged. After normalization, the statistical significance of differential miRNA expression was analyzed by *t*-test. Unsupervised hierarchical clustering and correlation analysis were performed with the miRNA data. Differentially expressed miRNAs were defined as those with expression in the MI oocytes for different treatments and differing by no less than 2- fold (*P*<0.05). We submitted our dataset to Gene Expression Omnibus and got the accession number(GSE57657, http://www.ncbi.nlm.nih.gov/geo/info/linking.html).

### Potential targets of differentially expressed miRNAs

Putative mRNA targets were predicted by three algorithms: DIANA, TargetScan 4.0 and PicTar. Only the targets identified by all three algorithms were analyzed further.

### Renilla luciferase reporter experiment

Before the transfection, 1×10^6^ 293T cells were inoculated with DMEM medium supplemented with 10% fetal bovine serum for 24 h in six-well plates. When the density of cells reached 50%–80%, cells were injected with 50 nM miR-133b mimic and miR-133b mimic negative control (Ribobio, Guangzhou, China) using 1×tiboFECT CP buffer and reagent (Ribobio), as well as 100 nM miR-133b inhibitor and miR-133b inhibitor negative control (Ribobio), according to the instructions of the manufacturer. Total RNAs and proteins were extracted 72 h after transfection and used for RT-PCR and Western blotting, respectively.

The 293T cells were injected with psiCHECK-2-NP, psiCHECK-3′ UTR and psiCHECK-3′ UTR-m, together with the different doses of miR-133b mimic using Lipofectamine2000 (Invitrogen Carlsbad, CA, USA), according to the manufacturer's instructions. Cells were collected 36 h after injection, and the ratios between Renilla and firefly luciferase were measured using Dual-Luciferase Reporter Assay System (E1910) (Promega, Madison, WI, USA).

### Immunofluorescence

Cells were plated on glass cover slips and washed with PBS. Then, cells were fixed with ice acetone for 5 min and washed three times. All fixed cells were treated with 0.3% hydrogen peroxide for 15 min at room temperature and again washed 3 times. Cells were blocked with 5% goat serum for 10 min, followed by removal of the serum. Cells were incubated with primary antibody (1∶1,000) overnight at 4°C; then incubated with FITC-labeled secondary antibody at room temperature for 20 min and washed 3 times, followed by analysis with confocal laser scanning microscopy.

### Western blotting

Following specified treatments, cells or tissues were washed twice with ice-cold PBS and harvested in sample buffer. Soluble extracts were prepared by centrifugation at 12000 g for 30 min at 4°C. Protein concentration was determined using the BCA kit (Pierce Chemical Co., Rockford, IL, USA). Equivalent amounts of protein (40 µg) for each sample were resolved in SDS-PAGE. After electrophoresis, proteins were transferred to nitrocellulose membranes. Membranes were incubated in TBST containing 5% nonfat milk for 1 h at room temperature. The blots were then reacted with primary antibody overnight at 4°C. Antibodies used for Western blotting analysis included TAGLN2 and β-actin antibody (Abcam, Cambridge, MA, USA). After washing with TBST, the membrane was then incubated with the corresponding horseradish peroxidase-conjugated secondary antibodies (Cell Signaling, Danvers, MA, USA). The signals were visualized by ECL (Pierce Chemical Co. Rockford, IL, USA) and then exposed to X-ray films.

### RNA extraction and real-time PCR

Because it was difficult to obtain more than one human oocyte at the same maturity stage in the same patient, all oocytes were tested individually while the RNA expression level was detected. In addition, it was technically challenging to normalize the reaction volume and composition if oocytes were transferred into the same reaction tube at different times.

Each RT reaction system contained two steps, primarily mixed 13ul reactions including 1 µg total RNA, 1 µl anchored-oligo(dT)18 primer, 2 µl randon hexamer primer, variable water for 10 min at 65°C, then added 4 µl 5×transcriptor reverse transcriptase transcriptase reaction buffer, 0.5 µl protector RNase inhibitor, 2 µl deoxynucleotide mix and 0.5 µl transcriptor reverse transcriptase(Transcriptor First Stand cDNA Synthesis Kit, roche,Guangzhou), followed on a ordered cycle (PCR system9700, ABI) 10 min at 25I) 10 miin at 55°C, 5 min at 85°C, and stored at −20°C.

Reactions for qRT-PCR occured with faststart universal SYBR green master ROX (roche) in the real-time PCR system (AB Stepone Plus). These reactions contained 25 µl 1× faststart universal SYBR green master ROX, 0.5 µl forward primer(30 µM), reverse primer(30uM), DEPC water and 5 µl template DNA, performed by denaturation 10 min at 95°C, followed by 40 cycles of 15 s at 95°C, 60 s at 58°C. For relative quantification at the single-cell level, it was problematic to choose a housekeeping gene that shows constant expression levels between individual samples.

Therefore, we used the relative quantification method with normalization to the cell number in this study. ΔCt was calculated using the following equation: ΔCt = Ct(calibrator)-Ct(test). The Ct value of the first sample was set as default calibrator value. The fold change was calculated using the following equation: Ratio (test/calibrator) = 2×[Ct(calibrator) - Ct(test)]. Unless otherwise indicated, all relative quantification was analyzed by this method.

Significance was defined according to *P*-values gained from two-tailed *t*-test analysis, or for single-cell ΔCt comparisons, one-tailed *t*-test analysis.

### Statistical analysis

Data are expressed as mean ± SD. The statistical significance of differences was assessed by Student's *t* tests or ANOVA, as appropriate; *P*<0.05 was considered statistically significant.
